# Bidirectional modulation of hyperalgesia via the specific control of excitatory and inhibitory neuronal activity in the ACC

**DOI:** 10.1186/s13041-015-0170-6

**Published:** 2015-12-02

**Authors:** Sukjae Joshua Kang, Chuljung Kwak, Jaehyun Lee, Su-Eon Sim, Jaehoon Shim, Taehyuk Choi, Graham L. Collingridge, Min Zhuo, Bong-Kiun Kaang

**Affiliations:** Department of Brain and Cognitive Sciences, College of Natural Sciences, Seoul National University, Seoul, 151-746 South Korea; Department of Biological Sciences, College of Natural Sciences, Seoul National University, Seoul, 151-747 South Korea; Interdisciplinary Program in Neuroscience, College of Natural Sciences, Seoul National University, Seoul, 151-747 South Korea; Centre for Synaptic Plasticity, School of Physiology and Pharmacology, University of Bristol, Bristol, BS8 1TD UK; Center for Neuron and Disease, Frontier Institutes of Life Science and of Science and Technology, Xi’an Jiaotong University, Xi’an, China; Department of Physiology, Faculty of Medicine, University of Toronto, 1 King’s College Circle, Toronto, ON M5S 1A8 Canada; Lunenfeld-Tanenbaum Research Institute, Mount Sinai Hospital, Ontario M5G 1X5, Toronto, ON M5S 1A8 Canada

## Abstract

Neurons in the anterior cingulate cortex (ACC) are assumed to play important roles in the perception of nociceptive signals and the associated emotional responses. However, the neuronal types within the ACC that mediate these functions are poorly understood. In the present study, we used optogenetic techniques to selectively modulate excitatory pyramidal neurons and inhibitory interneurons in the ACC and to assess their ability to modulate peripheral mechanical hypersensitivity in freely moving mice. We found that selective activation of pyramidal neurons rapidly and acutely reduced nociceptive thresholds and that this effect was occluded in animals made hypersensitive using Freund's Complete Adjuvant (CFA). Conversely, inhibition of ACC pyramidal neurons rapidly and acutely reduced hypersensitivity induced by CFA treatment. A similar analgesic effect was induced by activation of parvalbumin (PV) expressing interneurons, whereas activation of somatostatin (SOM) expressing interneurons had no effect on pain thresholds. Our results provide direct evidence of the pivotal role of ACC excitatory neurons, and their regulation by PV expressing interneurons, in nociception.

**Electronic supplementary material:**

The online version of this article (doi:10.1186/s13041-015-0170-6) contains supplementary material, which is available to authorized users.

## Background

The role of the anterior cingulate cortex (ACC) in pain conditions has been consistently demonstrated for the past several decades [1, 2]. Human brain imaging studies report responses of the ACC, and related cortical areas, to acute nociceptive stimuli [3–6]. Moreover, surgical lesions of the cingulate cortex (cingulotomy) reduced the unpleasantness of pain [7–10]. Given the important role of ACC in pain, numerous animal studies have been performed in this region to reveal the pathways and molecular mechanism of nociception and chronic pain [11–13]. For example, lesioning of rat ACC, similar to human cingulotomy, reduced aversive behavior during the conditioned place aversion test [14], and escape/avoidance test [15]. Numerous electrophysiological studies have also observed increased activation of the ACC in both acute and chronic pain situations. For example, an acute nociceptive stimulus in the peripheral region increased firing of ACC pyramidal neurons whereas a brush stroke had minimal effect [16–18]. Peripheral injury induced long-term potentiation (LTP) of excitatory synaptic responses in the ACC neurons [19, 20]. Pharmacological inhibition or genetic deletion of LTP related molecules in the ACC reduced pain in chronic models [21–24]. Furthermore, using pharmacological manipulations and electrical stimulation within the ACC, it has been shown that ACC activation can elicit aversive behaviors [25, 26]. Yet, the spatiotemporal precision of ACC neuronal activity in pain remains enigmatic and the function of different ACC neuronal subtypes is controversial.

Optogenetic tools are increasingly being used to identify the neural circuits underlying various types of behaviors or to reveal the functions of specific subtypes of cells [27, 28]. Several cre line mice are available for the selective manipulation of specific cell types [29, 30]. However, these methods have only just started to be applied to the understanding of pain pathways. Studies to date have been performed in peripheral regions [31], brain stem [32], medial prefrontal cortex (mPFC) [33–35] and ACC [36, 37]. The two optogenetic studies in the ACC used Thy1-ChR2 mice which expresses ChR2 indiscriminately within excitatory and inhibitory neurons. One group reported that activation of ACC showed anxiodepressive-like behaviors but no change in pain [36]. However, the other group observed an alleviation of acute inflammatory pain [37] which they attributed to the activation of inhibitory interneurons. The reason for these discrepant results is unknown.

In the present study, we used optogenetics and cre- expressing mouse lines to, for the first time, specifically manipulate the activity of excitatory neurons and interneurons within the ACC to establish whether specific neuronal subtypes within this brain region can acutely modulate nociceptive responses. We injected channelrhodopsin-2 (ChR2) or halorhodopsin (eNpHR3.0) carrying adeno-associated virus (AAV) in CaMKII-cre mice to activate or inhibit, respectively, the CAMKII-expressing ACC excitatory neurons. We also injected ChR2 into PV-cre or SOM-cre mice to activate specific PV positive or SOM positive interneurons. We found that activation of ACC pyramidal neurons decreased the basal mechanical threshold but did not further decrease the level in CFA-treated mice. In contrast, inhibition of these neurons reversed the effects of CFA treatment without affecting the basal mechanical threshold. Moreover activation of PV-type interneurons, but not SOM-type interneurons, also alleviated the CFA-induced pain hypersensitivity. These results show that excitatory neurons within the ACC are both necessary and sufficient for nociceptive processing and that they are under tight regulation by PV-expressing interneurons. These results suggest that ACC excitatory neurons are one of the critical mediators of nociception.

## Methods

### Animals

Adult (8–12 week old) male CaMKII-, PV- or SOM- cre mice (Jackson Laboratory) were used. All animals were housed under a 12 h light/dark cycle with food and water provided *ad libitum*. All works were conducted according to the policy and regulation for the care and use of laboratory animals approved by Institutional Animal Care and Use Committee at Seoul National University.

### Stereotaxic virus injection, optic cannula implantation

AAV carrying DIO-ChR2-EYFP, DIO-eNPHR3.0-EYFP or DIO-EYFP constructs was injected into 6 week-old CaMKII-cre hetero male mice. PV-cre hetero and SOM-cre hetero or wild-type littermates (control) were also injected with AAV-DIO-ChR2-EYFP. Mice were anesthetized with an intraperitoneal injection of ketamine-xylazine (0.1 mg per gram body weight ketamine, 0.01 mg per gram body weight xylazine) and the head was fixed in a stereotaxic apparatus (Kopf Instruments, Tujunga, CA, USA). A small craniotomy was performed and, five holes were drilled. Two holes were drilled bilaterally between the hippocampus and cerebellum for screw implantation. The other three holes were drilled for virus injection and optic cannula implantation in the ACC. The virus was delivered using a 10 μl syringe (Hamilton) and a 30 gauge metal needle. The injection volume and flow rate (0.5 μl at 0.1 μl/min) were controlled by an injection pump (WPI). Virus was injected into both side of the ACC (anteroposterior [AP] + 1.0 mm from bregma, mediolateral [ML] ± 0.35 mm, dorsoventral [DV] −2.2 mm). After injection, the needle was left for additional 7 min and then was slowly removed. The optic cannula (MFC_200/230-0.39_2mm_ZF1.25_FLT, Doric Lenses., Quebec, Canada) was implanted between the virus injection site (AP + 1.0 mm from bregma, ML + 0 mm, DV – 1.25 mm). The optic cannula was then fixed with dental cement.

### Behavior

#### Electrical von Frey system for checking the mechanical threshold

A pre-test was done 3 weeks after the surgery and CFA (1:1 mixture with saline) or saline 10 μl was injected in the hind paw after the test. 3 days later the post-test was performed. In each test, there were three light ‘off’ and two light ‘on’ sessions (off1-on1-off2-on2-off3, 5 min intersession interval). Paw withdrawal threshold was measured with the electronic von Frey system. The averages of three values were calculated as the threshold of that session. 593 nm light was given continuously in an intensity of 7–9 mW/cm^2^. 473 nm light was given at 10 Hz (40 ms pulse) throughout the ‘on’ period for the CaMKII-cre mice. PV-cre and SOM-cre mice were given 473 nm light in 300 ms pulses with 2 s inter-pulse intervals. An intensity of 20–25 mW/cm^2^ at fiber tip was used in all ChR2 experiments.

#### Open field test

Mice were exposed for 15 min to the open field apparatus under dim light. The open field was a square opaque white box (40 × 40 × 40 cm) in which mice were monitored with a tracking program (EthoVision 3.1, Noldus, Netherlands). Light stimulation was given throughout the experiment.

### Electrophysiology

#### Patch clamp recording

For whole-cell patch clamp recording, 300 μm thick coronal slices of ACC were prepared with a Leica VT-1000S slicer and incubated in artificial cerebrospinal fluid (aCSF) at 25 ~ 26 °C for 1 h. The aCSF comprised 124 mM NaCl, 2.5 mM KCl, 1 mM NaH_2_PO_4_, 25 mM NaHCO_3_, 10 mM Glucose, 2 mM CaCl_2_, 2 mM MgSO_4_ was used for the incubation and bath solution. The bath solution was oxygenated with 95 % CO_2_, 5 % O_2_ mixed gas and perfused 2 ml/min at 25 ~ 26 °C (TC-324B, Warner). The slices were transferred to the recording chamber of a BX51WI microscope (Olympus) and visualized with a ProgRes MFcool CCD camera.

For current-clamp recording, the borosilicate glass recording pipettes were filled with internal solution containing 145 mM K-gluconate, 5 mM NaCl, 0.2 mM EGTA, 10 mM HEPES, 2 mM MgATP, 0.1 mM Na_3_GTP, 1 mM MgCl_2_ (pH 7.2 with KOH, 280 ~ 290 mOsm). For current injection, pyramidal neurons in layer 2/3 were current clamped and 20 pA-140 pA depolarizing current was injected for 10 s while 473 nm light was delivered to the ACC area using a 300 ms pulse width and 2 s inter-pulse interval. For pulse train experiments, pyramidal neurons in layer 2/3 were current clamped and 2 Hz depolarizing current pulses (80 pA pulse with 200 ms width) were injected for 90 s. 473 nm light was delivered to the ACC area using 300 ms pulses with a 2 s inter-pulse interval for 30 s in the middle of the trains.

#### In-vivo recording

After 3 weeks of recovery, the mice was anesthetized with urethane (Sigma, 0.13 g/100 g) injection and the head was fixed in a stereotaxic apparatus (Kopf Instruments, Tujunga, CA, USA). A small craniotomy was performed and two holes were drilled above the ACC on each side of the brain (AP + 1.0 mm from bregma, ML ± 0.0 mm) and a third was drilled in the cerebellum for reference. A customized optrode, composed of a tungsten electrode (A-M Systems, USA) and optic cannula (MFC_200/230-0.39_50mm_ZF1.25_FLT, Doric lenses, USA), was inserted into the ACC for multiunit recording. Neural activity was amplified 1000 fold and digitized (sampled at 32 kHz, filtered at 600–6000 Hz) using a Digital Lynx data acquisition system (Neuralynx).

### Data analysis

All data are presented as mean ± SEM. Statistical comparisons were made using the *t*-test, one-way and two-way ANOVA by SigmaPlot 11.0. The post hoc Bonferroni test was used for further comparison. If the data did not pass the equal variance test, one way ANOVA was done in ranks and Dunn’s method was used for post-hoc test. In all cases, statistical significance was indicated by *p < 0.05.

## Results

### Discrete cellular population in the ACC and manipulation of the excitatory neurons

**Fig. 1:**
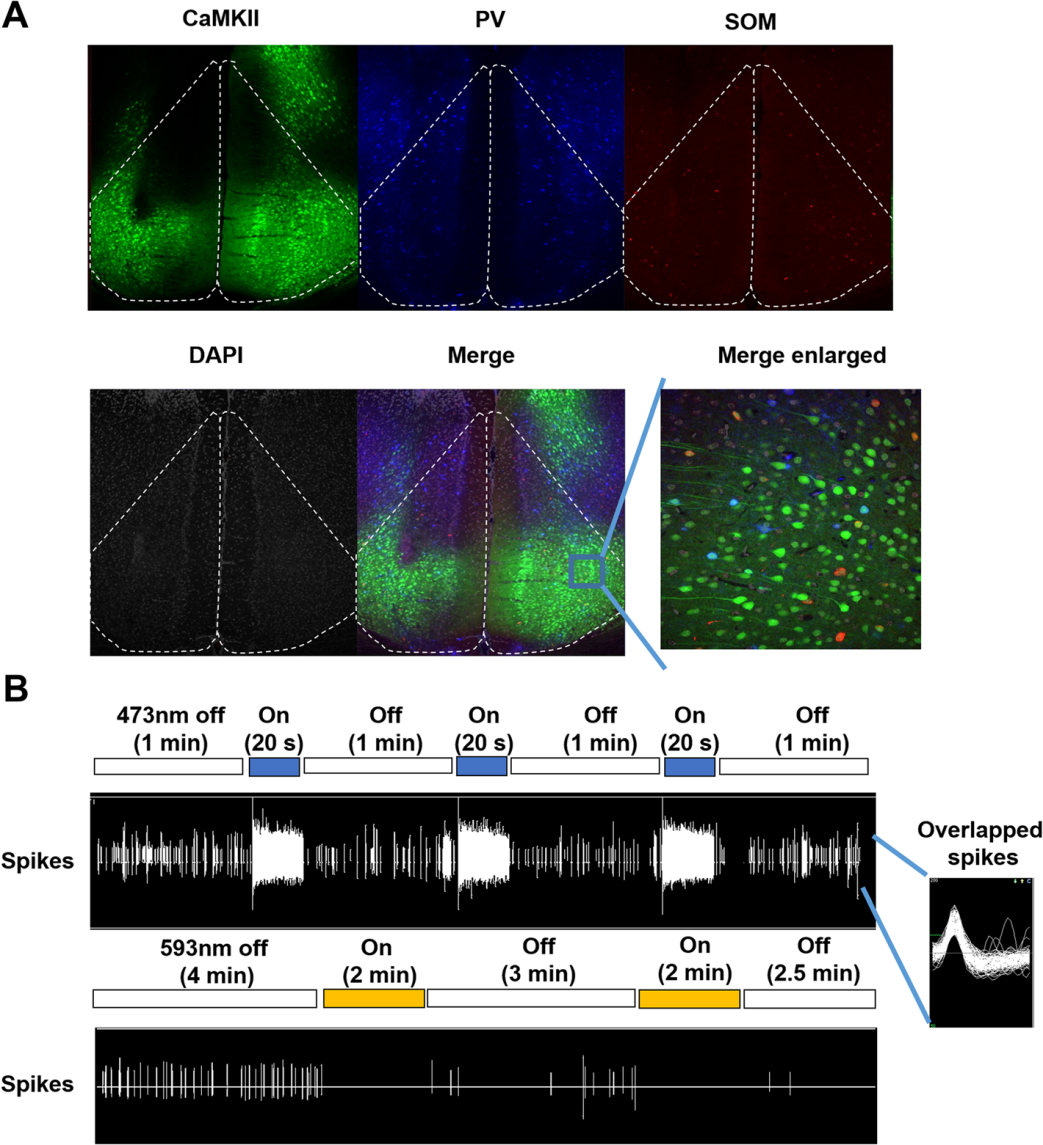
Distinct cell populations and viral expression of rhodopsins in the ACC of CaMKII-cre mice. **a** Tissue staining of three different markers in the ACC. AAV-DIO-EYFP was injected to ACC of CaMKII-cre mice. CaMKII (EYFP), PV and SOM do not co-localize in the ACC. Dotted polygons indicate one side of ACC. **b** Optogenetic modulation during multiunit in vivo recording in the ACC of anesthetized mice. 473 nm light stimulated the basal firing of CaMKII-cre mice injected with AAV-DIO-ChR2-EYFP and 593 nm light inhibited the basal firing of CaMKII-cre mice injected with AAV-DIO-eNpHR-EYFP

In the present study, we used three cre mouse lines (CaMKII-, PV-, SOM-cre) for optogenetic manipulation in the ACC. We performed immunostaining in the ACC to observe the distribution of these three different molecular targets. AAV-DIO-EYFP was injected bilaterally into the ACC of CaMKII-cre mice and PV and SOM were stained with antibodies (Fig. 1a, Additional file 1: Figure S1). All three markers were expressed throughout the ACC but within discrete cellular populations.

We next tested the effectiveness of the rhodopsins in pyramidal neurons within the ACC. AAV-DIO-eNpHR3.0, AAV-DIO-ChR2 or AAV-DIO-EYFP were injected into the ACC of CaMKII-cre mice and multiunit in vivo recording using an optrode was performed 3 weeks later. Activation of ChR2 with blue (473 nm) light stimulated the ACC neurons whereas activation of eNpHR3.0 with yellow (593 nm) light inhibited their activity (Fig. 1b).

### Activation of excitatory neurons in the ACC decreases the mechanical threshold

**Fig. 2:**
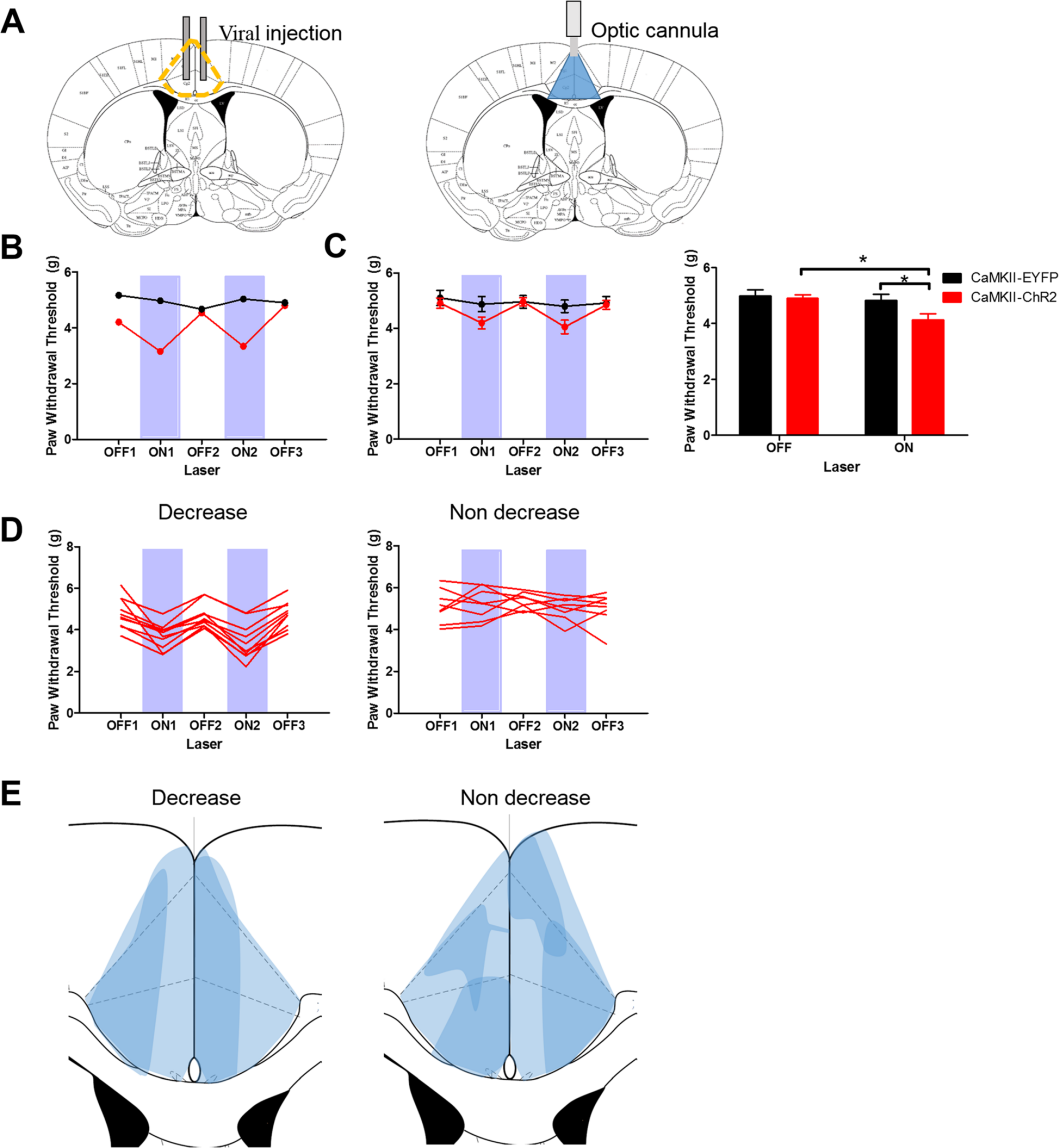
Activation of CaMKII-positive neurons in the ACC decreases the mechanical threshold. **a** Schematic diagram of viral injection site (left) and optic cannula placement (right) in the ACC. **b** One example of the effects of blue light in a CaMKII-ChR2 expressing mouse (red) and an EYFP expressing mouse (black) in the von Frey test. **c** Pooled data for ChR2 and EYFP mice. On average there was a reduction in the mechanical threshold in the ChR2 group. The graph on the right plots the combined data for the two ON and three OFF episodes. There was a significant effect of laser light in the ChR2 group: Off: 4.90 ± 0.13 g, on: 4.12 ± 0.22 g; *n* = 18) but not in the EYFP group (off: 4.99 ± 0.22 g, on: 4.81 ± 0.22 g; *n* = 10) [*p* = 0.011 in virus and light interaction, two-way repeated measures ANOVA]. **d** Not all animals showed a decrease of the mechanical threshold during light activation. Out of 18 animals, 10 showed a decrease whilst 8 did not. **e** The overlapped pattern of ChR2 expression in each group

Throughout our study, we injected AAV-DIO-ChR2, AAV-DIO-eNpHR3.0 or AAV-DIO-EYFP in the ACC bilaterally and the optic cannula was implanted in the middle of the viral injection sites (Fig. 2a). We first tested whether specific activation of ACC excitatory neurons is itself sufficient to modulate the nociceptive responses. To address this question, AAV-DIO-ChR2 was injected into the ACC of CaMKII-cre mice (CaMKII-ChR2) and 473 nm light was pulsed at 10 Hz throughout the ‘ON’ period for ACC activation. This frequency was selected according to the previous in vivo whole-cell patch recording data in the ACC during noxious stimulation [16]. Interestingly, the mechanical threshold of the majority (10/18) of the mice decreased when the light was given and fully recovered back to the baseline value when the light was turned off (Fig. 2b, d). This effect was replicated when a second period of light excitation was employed (Fig. 2b, d). In contrast, the AAV-DIO-EYFP injected control group was invariably unaffected by light (Fig. 2b) [EYFP (off: 4.99 ± 0.22 g, on: 4.81 ± 0.22 g; *n* = 10) and ChR2 (off: 4.90 ± 0.13 g, on: 4.12 ± 0.22 g; *n* = 18; Fig. 2c)]. The ‘off’ and ‘on’ sessions were averaged for statistical analysis and there was a statistically significant interaction between light (‘off’ and ‘on’) and virus (‘ChR2’ and ‘EYFP’) infused (*p* = 0.011, two-way repeated measures ANOVA). There was no difference in the patterns of ChR2 expression between the “decrease” and “non-decrease” groups (Fig. 2e).

### Activation of ACC excitatory neurons has no effect in chronic pain conditions

**Fig. 3:**
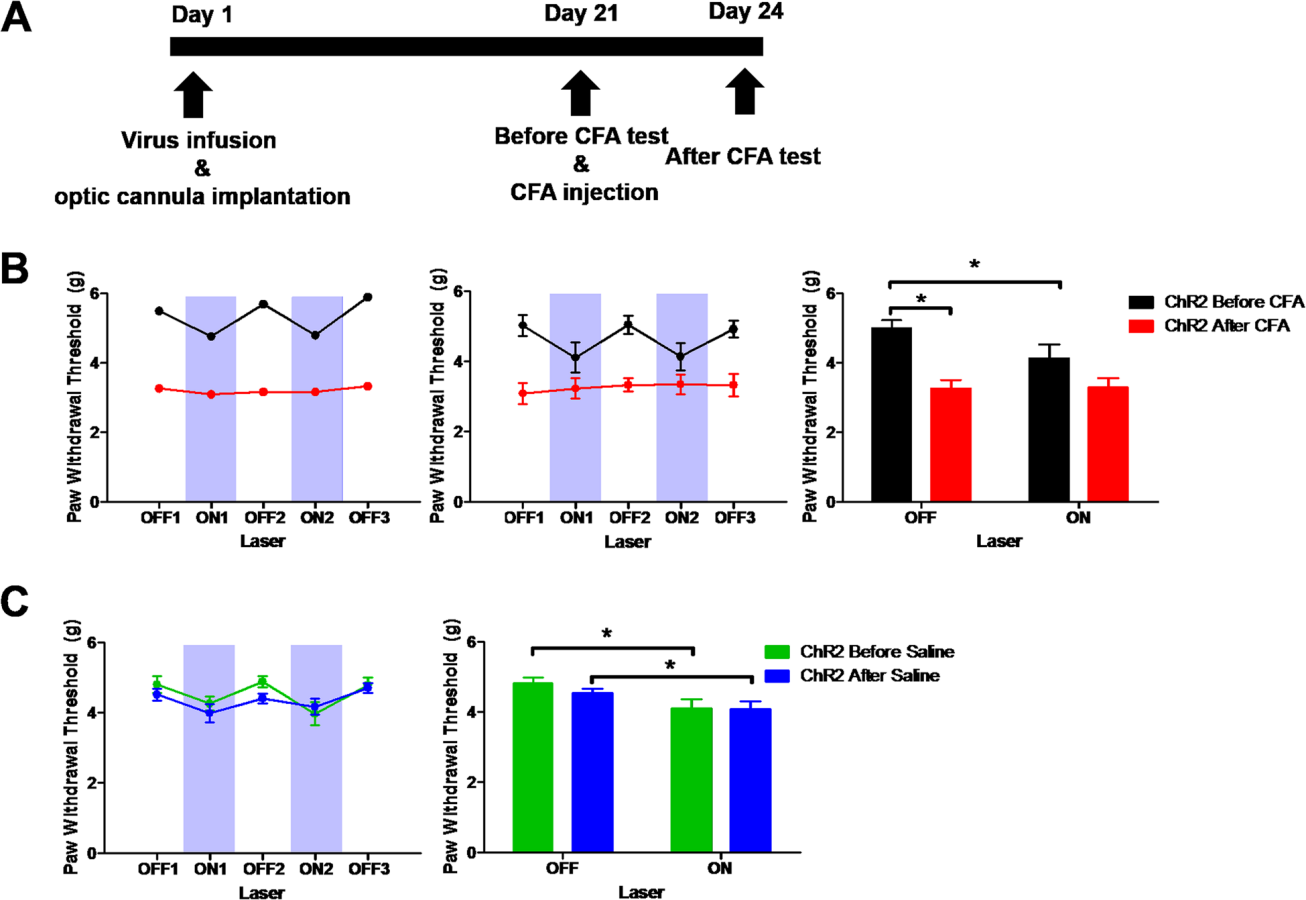
Activation of CaMKII-positive neurons in the ACC has no effect on the mechanical threshold in CFA-treated mice. **a** Schematic of the behavioral experiment. The pre-test was performed three weeks after the virus infusion and optic cannula implantation. After the pre-test, CFA or saline was injected into the sole of the right hind paw. The post-test was done three days after the CFA injection. **b** Effects of blue light in a ChR2 mouse (left) and pooled data (right) before (black) and after (red) CFA treatment. There was a significant effect of 473 nm laser before CFA (off: 5.00 ± 0.23 g, on: 4.13 ± 0.40 g; *n* = 8). CFA injection resulted in a lowering of the mechanical threshold and blue light had no effect (off: 3.25 ± 0.25 g, on: 3.29 ± 0.28 g; *n* = 8) [*p* < 0.001 in CFA, *p* = 0.108 in laser, *p* = 0.120 in CFA and light interaction, two-way repeated measures ANOVA]. **c** Equivalent results for the saline treated group. There was a significant effect in both the pre-test (off: 4.83 ± 0.15 g, on: 4.12 ± 0.25 g; *n* = 10) and post-test (off: 4.54 ± 0.11 g, on: 4.08 ± 0.23 g; *n* = 10) [*p* = 0.128 in saline, *p* = 0.027 in laser, *p* = 0.445 in saline and light interaction]

To examine the role of ACC excitatory neurons in chronic pain condition, we used CFA treatment and performed behavioral experiments as shown schematically in Fig. 3a. In contrast to the effects of activating ACC excitatory neurons of CaMKII-ChR2 mice in a non- painful situation (Before CFA; off: 5.00 ± 0.23 g, on: 4.13 ± 0.40 g; *n* = 8; Fig. 3b black), there was no modification of the mechanical threshold in the chronic pain state (After CFA; off: 3.25 ± 0.25 g, on: 3.29 ± 0.28 g; *n* = 8; Fig. 3b red). This suggests an occlusion of the effects of optogenetic activation of excitatory ACC neurons and the effects of inflammatory pain-induced signals. In the saline injected control group, there was a similar light-induced reduction in the pain threshold both before (off: 4.83 ± 0.15 g, on: 4.12 ± 0.25 g; *n* = 10; Fig. 3c green) and after saline (off: 4.54 ± 0.11 g, on: 4.08 ± 0.23 g; *n* = 10; Fig. 3c blue) injection. There was no light effect in control groups (Additional file 2: Figure S2).

To test the effects of ChR2 activation on locomotion and anxiety we performed an open field test (OFT). We found that there were no difference between the CaMKII-EYFP and CaMKII-ChR2 groups in the OFT when light was given throughout the experiment. (Additional file 4: Figure S4A). These results identify excitatory ACC neurons as effectors of pain responses and show that their activation alone is sufficient for a significant alteration in the mechanical pain threshold.

### Inhibition of excitatory neurons in the ACC reverses the effects of inflammatory pain

**Fig. 4:**
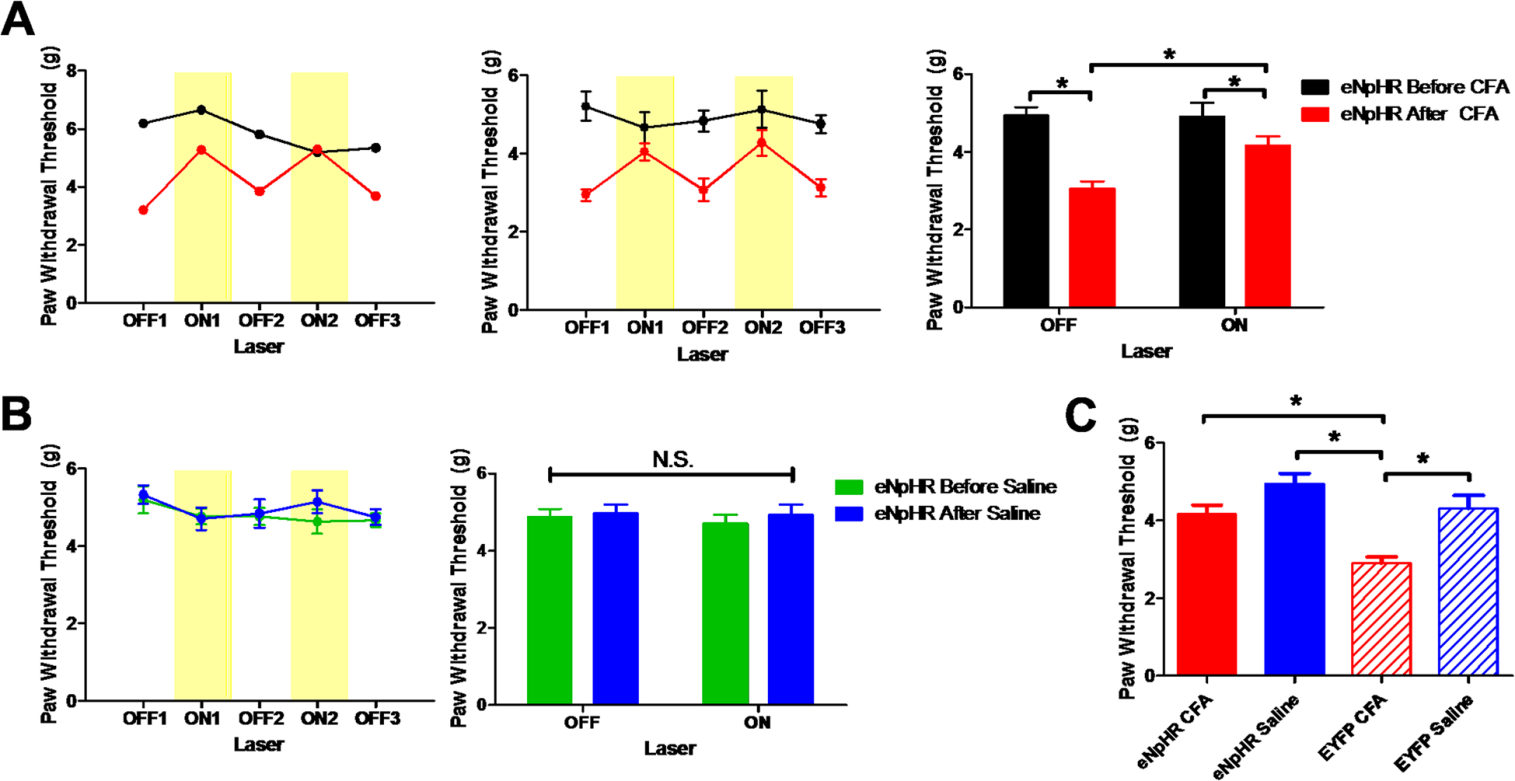
Inhibition of CaMKII-positive neurons in the ACC alleviates the CFA-induced decrease of the mechanical threshold. **a** Results of the eNpHR-CFA group. There was no effect of yellow light before CFA (off: 4.94 ± 0.22 g, on: 4.90 ± 0.36 g; *n* = 9). However, the mechanical threshold was increased when light was given after CFA injection (off: 3.05 ± 0.19 g, on: 4.16 ± 0.24 g; *n* = 9) [*p* = 0.011 in CFA and laser interaction, two-way repeated measures ANOVA]. **b** Result of eNpHR-Saline group. There was no light effect before (4.88 ± 0.21 g, on: 4.71 ± 0.24 g; *n* = 7) and after saline (off: 4.98 ± 0.23 g, on: 4.93 ± 0.27 g; *n* = 7). **c** Comparison of the post-test light ‘on’ period in all four groups. There was statistically significant difference between groups (*p* < 0.001, one-way ANOVA)

We next inhibited ACC excitatory neurons, expressing eNpHR3.0 (CaMKII-eNpHR), with yellow light and determined the mechanical threshold before and 3 days after CFA treatment. Interestingly, optogenetic illumination had no effect on the mechanical threshold before CFA injection (off: 4.94 ± 0.22 g, on: 4.90 ± 0.36 g; *n* = 9) but after CFA treatment it reversed the mechanical pain threshold to near control values (3.05 ± 0.19 g, on: 4.16 ± 0.24 g; *n* = 9; Fig. 4a). The effect of optogenetic illumination was fully reversible and repeatable. Thus, optogenetic inhibition of excitatory neurons in the ACC substantially reversed the increase of mechanical sensitivity in the chronic inflammation pain model (*p* = 0.011 in drug and light interaction, two-way ANOVA repeated measures). In contrast, there was no effect of light in the saline injection group and other control groups (Fig. 4b, Additional file 3: Figure S3 ). Comparison of the light ‘ON’ phases for all four groups showed statistically significant differences between the groups (*p* <0.001, one-way ANOVA), such that the EYFP-CFA group was significantly different from the other groups (Fig. 4c). To test whether the inhibition affects locomotion and anxiety we performed the OFT but could not see any difference between CaMKII-EYFP and CaMKII-eNpHR groups (Additional file 4: Figure S4B).

To test for the specificity of excitatory neurons within the ACC for the modulation of the pain threshold, we examined the effects of light on CaMKII-eNpHR neurons within the restrospenial cortex (RSG), a region adjacent to the ACC. In contrast to the ACC, yellow light had no effect on pain thresholds (Additional file 5: Figure S5). These result shows that transient inhibition of excitatory neurons in the ACC can significantly reverse the enhanced sensitivity to a mechanical stimulus caused by inflammation. This suggests, therefore, that excitatory neurons in the ACC may be effectors of the sensation of pain.

### Optogenetic activation of inhibitory neurons in the ACC reverses the effects of inflammatory pain

**Fig. 5:**
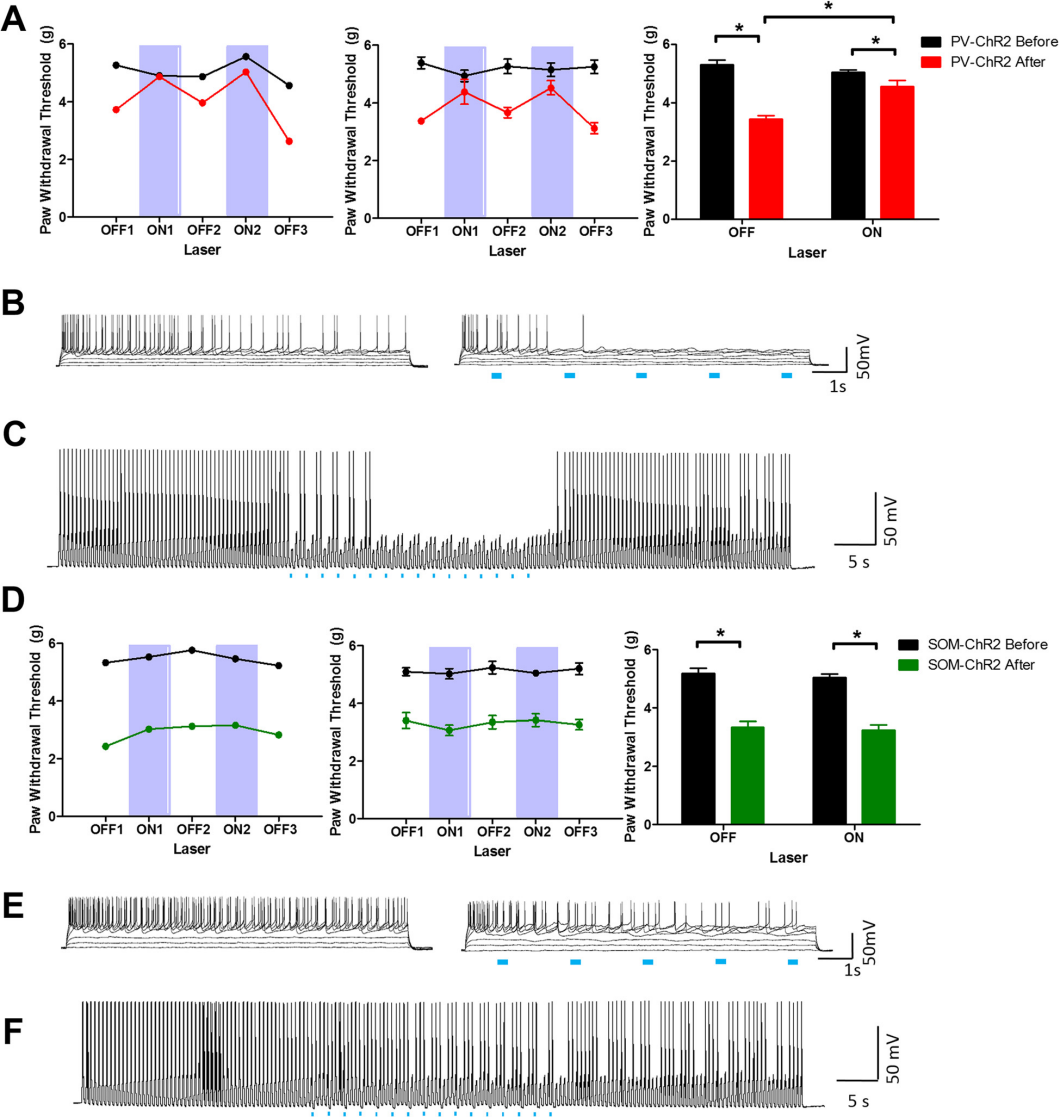
Activation of PV-positive interneurons in the ACC alleviates the CFA-induced decrease of the mechanical threshold. **a** Results of the PV-ChR2 CFA group. There was no effect of blue light before (black) CFA (off: 5.30 ± 0.15 g, on: 5.04 ± 0.07 g; *n* = 6) however, there was significant effect after (red) CFA injection (off: 3.44 ± 0.11 g, on: 4.55 ± 0.21 g; *n* = 6) [*p* = 0.002 in CFA and laser interaction, two-way repeated measures ANOVA]. **b** Result of current injection experiment with PV-ChR2 mice. A pyramidal neuron in the ACC was current clamped and current was injected without (left) and with blue light (right). Blue dots indicate illumination periods. Firing rates of the pyramidal neurons decreased when light was given. **c** Result of pulse train experiment with PV-ChR2 mice. A pyramidal neuron in the ACC was current- clamped and current pulses were given for 90 s while light was given for 30 s as indicated by the blue dots. Firing of the pyramidal neurons decreased as a result of illumination. **d** Result of the SOM-ChR2 CFA group. There was no light effect either before (off: 5.11 ± 0.15 g, on: 4.95 ± 0.11 g; *n* = 12) or after CFA injection (off: 3.32 ± 0.16 g, on: 3.30 ± 0.13 g; *n* = 12). **e** Result of current injection experiment with SOM-ChR2 mice. A pyramidal neuron in the ACC was current-clamped and current was injected without (left) and with blue light (right). Blue dots indicate light on periods. Light had only a small effect on the firing of pyramidal neurons. **f** Result of pulse train experiment with SOM-ChR2 mice. A pyramidal neuron in the ACC was current- clamped and current pulses were given for 90 s while light was illuminated for 30 s, as the blue dots indicate, but had only a small effect on firing rate

The ACC comprises both excitatory neurons and a diverse array of inhibitory neurons that may play distinct roles in the modulation of pain thresholds. A major class of GABAergic inhibitory neurons expresses parvalbumin (PV). We therefore tested whether activation of the ACC PV-positive interneurons could modulate the mechanical threshold during chronic inflammation pain. 473 nm light was given at 0.5 Hz throughout the ON period, using a protocol previously applied to the amygdala [30]. We found that activation of PV-ChR2 expressing neurons could substantially alleviate CFA-induced pain. In the PV-ChR2 group there was no significant effect of the 473 nm light before CFA (off: 5.30 ± 0.15 g, on: 5.04 ± 0.07 g; *n* = 6). However, there was significant increase in the mechanical threshold after CFA injection (off: 3.44 ± 0.11 g, on: 4.55 ± 0.21 g; *n* = 6; Fig. 5a; *p* = 0.002 in CFA and light interaction, two-way repeated measures ANOVA). Light had no effect in other control groups (Additional file 6: Figure S6). Since the behavioral outcome is similar to that of the CaMKII-eNpHR mice, we wondered whether PV optogenetic activation will inhibit excitatory neurons. Current injection and pulse train tests were performed using whole-cell patch-clamp recording and light was given at 0.5 Hz to mimic the behavioral experiments with the PV-ChR2 mice. In the current injection experiment, (Fig. 5b, right), excitatory neurons showed a greater decrease of firing in the light group compared to the control group (Fig. 5b, left). Moreover, in the pulse train test, the firing of excitatory neurons were reduced during the light presentation and recovered when the illumination was stopped (Fig. 5c). These results demonstrate that optogenetic excitation of PV+ interneurons inhibits the activity of ACC pyramidal neurons.

In contrast to the effects of activation of PV+ interneurons, there was no effect of the illumination of SOM-ChR2 expressing neurons on the mechanical threshold either before (off: 5.11 ± 0.15 g, on: 4.95 ± 0.11 g; *n* = 12) or after CFA treatment (off: 3.32 ± 0.16 g, on: 3.30 ± 0.13 g; *n* = 12; Fig. 5d, Additional file 7: Figure S7). The whole-cell patch-clamp experiments also showed minimal effect of optogenetic excitation of SOM+ interneurons on the activity of ACC pyramidal neurons (Fig. 5e, f). These results show that the selective activation of PV+ interneurons, but not SOM+ interneurons, in the ACC is able to reverse the effects of inflammation on the mechanical pain threshold, without affecting the mechanical pain threshold under basal conditions.

## Discussion

In the present study, we have demonstrated that neurons within the ACC are acutely involved in the nociceptive responses of painful stimuli. We found that the specific activation of excitatory neurons in the ACC results in a lowering of the mechanical pain threshold, which is consistent with the view that the ACC is important in nociceptive responses [1, 2, 12, 16]. Interestingly, the effect occurred rapidly upon optogenetic excitation, was rapidly reversible upon termination of illumination and was reproducible upon a second trial. This strongly suggests that these ACC neurons are effectors of the nociceptive responses as opposed to being a secondary reporter of the stimulus. This may be due to activating the descending facilitation projection [38]. Of course, our findings do not exclude the possibility that there are other primary mediators of the nociceptive responses. However, the observation that simply exciting a proportion of excitatory neurons in the ACC was able to significantly modify the mechanical pain threshold demonstrates the key role played by these neurons.

The observation that optogenetic excitation of excitatory ACC neurons was unable to further lower the mechanical pain threshold in mice treated with CFA suggests an occlusion between the two effects. In other words, an increase in activity in these ACC neurons may be mediating the nociceptive responses of inflammatory pain. If this is the case, the prediction would be that optogenetic inhibition of this neuronal may reverse the effects of inflammation on the mechanical pain threshold. The finding that optogenetic inhibition of excitatory ACC neurons had a substantial effect on the mechanical threshold after CFA treatment strongly suggests that these neurons do indeed convey this nociceptive signal. Interestingly, optogenetic inhibition of these neurons had no effect under normal (non-inflammatory) conditions. Thus, an increase in activity of this neuronal population signals the hyperalgesic state. When considered together these results imply that excitatory neurons in the ACC are both necessary and sufficient to mediate sensitization to mechanical stimuli.

Excitatory neurons in the cortex are under strong regulation by GABAergic interneurons. We found that optogenetic stimulation of one class, PV-containing interneurons, was sufficient to have a substantial effect on the pain threshold. This effect was again specific for the inflamed state. Indeed, in all respects, stimulation of this single interneuron class mimicked the effects of inhibition of the excitatory neurons within this brain structure. This observation is consistent with the role of PV+ interneurons as mediators of powerful feed-forward inhibition of pyramidal neurons [39]. It shows that excitatory ACC neurons are under powerful influence of this interneuronal subclass during painful stimuli and that their specific activation is sufficient to alleviate mechanical hypersensitivity, presumably by their inhibition of pyramidal neurons in the ACC. Recently, an opposite conclusion has been reached with respect to the role of the mPFC, especially the prelimbic cortex (PL), in the processing of painful information. Activation of PL excitatory neurons reduced pain [34], and activation of PV interneurons induced more pain [35]. Moreover, activation of PL projection to nucleus accumbens relieved pain [33]. These results highlight how anatomically distinct cortical regions have discrete roles in pain processing.

Recently two other studies have used optogenetic approaches to address specifically the functional roles of the ACC but they drew different conclusions. In one study it was reported that optogenetic stimulation of Thy1-ChR2 mice did not affect the mechanical threshold [36] whereas in the other study it was reported that activation of Thy1-ChR2 mice resulted in analgesia [37]. Why the two studies reached different conclusions is unclear, though it may be due to the use of different Thy1-ChR2 mice lines. There are several possibilities why our conclusions differs from the study of Barthas et al. [36]. Firstly, in the Thy1 mouse there is expression of ChR2 in multiple neuronal types. Indeed, we found that specific activation of ChR2 in pyramidal neurons and PV+ interneurons had diametrically-opposed effects. Secondly, in this previous study, optogenetic stimulation and the mechanical threshold test were not performed at the same time. Therefore, this raises the possibility that chronic activation of ACC excitatory neurons does not affect the mechanical threshold but rather this requires their acute activation. Our study does, however, agree with the report of Gu et al. [37], which attributed the analgesia to the activation of inhibitory neurons, and extends these findings by identifying PV+ neurons as a critical inhibitory neuronal class.

In the study by Barthas et al. [36] it was reported that optogenetic stimulation resulted in a long lasting alterations in anxiety and depressive-like behaviors [36]. In the present study we did not observe any effects of optogenetic manipulation on anxiety-like behavior, as assessed using the open field test. This difference could be explained by the need for repeated stimulation to elicit mood-related behaviors and/or because in the Thy1-ChR2 mice mixed cell types are affected.

## Conclusions

In conclusion, we have identified two specific neuronal types within the ACC that are involved in the nociceptive responses to inflammatory pain. These results raise the possibility that specifically targeting these neuronal populations may lead to effective treatments of painful states.

## Additional files

Additional file 1: Figure S1. ACC neuronal population. Enlarged image of stained ACC slice. CaMKII, PV and SOM do not co-localize. (PNG 2291 kb) Additional file 2: Figure S2. Control data of optogenetic activation on ACC CaMKII-positive neurons. A, Result of the CaMKII-EYFP-CFA group. There was no light effect during either the pre-test (off: 5.18 ± 0.17 g, on: 4.96 ± 0.19 g; *n* = 7) or the post-test (off: 3.15 ± 0.12 g, on: 3.15 ± 0.14 g; *n* = 7). There was only statistically significant effect of CFA treatment *per se* (*p* < 0.001, two-way repeated measures ANOVA). B, Result of CaMKII-EYFP-Saline group. There was no light effect either before (off: 4.77 ± 0.44 g, on: 4.63 ± 0.44 g; *n* = 6) or after saline injection (off: 4.96 ± 0.17 g, on: 4.82 ± 0.22 g; *n* = 6). (PNG 45 kb) Additional file 3: Figure S3. Control data of optogenetic inhibition on ACC CaMKII-positive neurons. A, Result of the CaMKII-EYFP-CFA group. There was no light effect before (off: 4.77 ± 0.20 g, on: 4.97 ± 0.15 g; *n* = 6) or after CFA (off: 2.81 ± 0.22 g, on: 2.90 ± 0.16 g; *n* = 6). There was only a statistically significant effect of CFA treatment *per se* (*p* < 0.001, two-way repeated measures ANOVA). B, Result of CaMKII-EYFP-Saline group. There was no light effect either before (off: 4.44 ± 0.30 g, on: 4.17 ± 0.28 g; *n* = 5) or after CFA (off: 4.27 ± 0.22 g, on: 4.31 ± 0.24 g; *n* = 5). (PNG 42 kb) Additional file 4: Figure S4. Control experiments for locomotion and anxiety. A, Results of the CaMKII-EYFP and CaMKII-ChR2 groups in the open field test (OFT). There was no difference between the two groups in either locomotion (EYFP: 5824 ± 457.6 cm, *n* = 8; ChR2: 6207 ± 244.8 cm, *n* = 17; *p* = 0.4273, Unpaired *t* test) or time spent in the center (EYFP: 9.17 ± 1.85 %, *n* = 8; ChR2: 11.27 ± 1.68 %, *n* = 17; *p* = 0.4559, Unpaired *t* test). B, Results of the CaMKII-EYFP and CaMKII-eNpHR groups in the OFT. There was no difference between the two groups in either locomotion (EYFP: 6616 ± 278.4 cm, *n* = 7; eNpHR: 6043 ± 212.9 cm, *n* = 12; *p* = 0.1209, Unpaired *t* test) or time spent in the center (EYFP: 10.96 ± 2.34 %, *n* = 7; eNpHR: 10.15 ± 1.35 %, *n* = 12; *p* = 0.7488, Unpaired *t* test). (PNG 28 kb) Additional file 5: Figure S5. Optogenetic inhibition of CaMKII-positive neurons in the retrospenial cortex (RSG). There was no light effect either before (off: 5.07 ± 0.07 g, on: 5.29 ± 0.18 g; *n* = 8) or after CFA injection (off: 3.28 ± 0.13 g, on: 3.38 ± 0.19 g; *n* = 8). (PNG 21 kb) Additional file 6: Figure S6. Control data of optogenetic activation of ACC PV-positive interneurons. A, Result of the PV-ChR2 saline group. There was no light effect either before (off: 5.09 ± 0.22 g, on: 4.84 ± 0.09 g; *n* = 6) or after saline injection (off: 5.18 ± 0.18 g, on: 4.79 ± 0.23 g; *n* = 6). B, Result of the WT-ChR2 CFA group. There was no light effect before (off: 5.18 ± 0.13 g, on: 5.12 ± 0.12 g; *n* = 8) or after CFA (off: 3.33 ± 0.12 g, on: 3.58 ± 0.19 g; *n* = 8). There was only statistically significant effect of CFA treatment *per se* (*p* < 0.001, two-way repeated measures ANOVA). C, Result of the WT-ChR2 saline group. There was no light effect either before (off: 4.7 ± 0.15 g, on: 4.93 ± 0.28 g; *n* = 5) or after saline (off: 4.94 ± 0.15 g, on: 4.67 ± 0.27 g; *n* = 5). (PNG 70 kb) Additional file 7: Figure S7. Control data of optogenetic activation of ACC SOM-positive interneurons. Result of WT-ChR2 CFA group. There was no light effect either before (off: 5.15 ± 0.20 g, on: 5.32 ± 0.20 g; *n* = 6) or after CFA injection (off: 3.10 ± 0.09 g, on: 3.24 ± 0.07 g; *n* = 6). (PNG 23 kb)
