# Supplementary figures and images for: Bidirectional modulation of hyperalgesia via the specific control of excitatory and inhibitory neuronal activity in the ACC
Source: Mol Brain. 2015 Dec 2;8:81. doi: 10.1186/s13041-015-0170-6 (PMC4668615; doi:10.1186/s13041-015-0170-6)

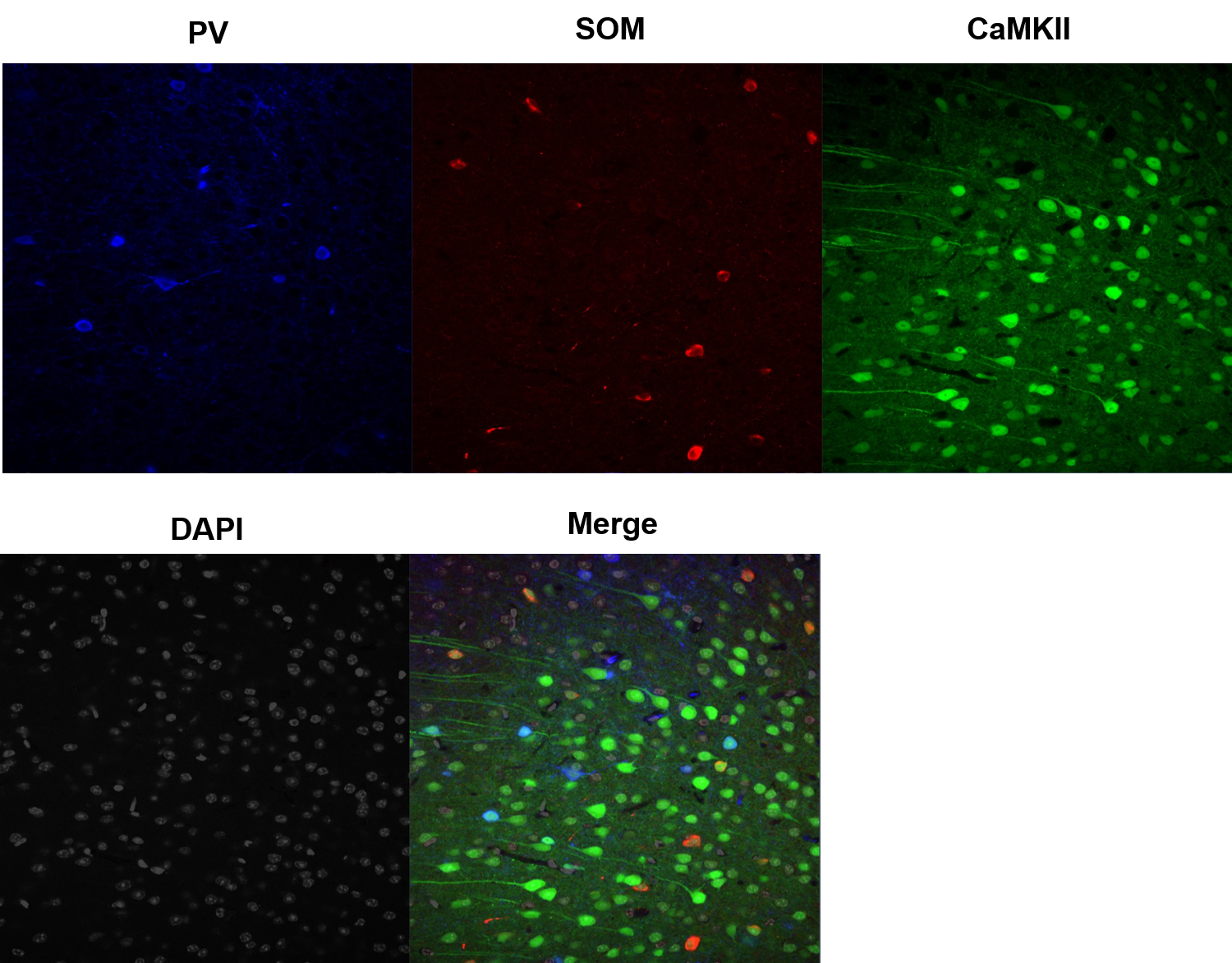

Supplement: Additional file 1: Figure S1. — ACC neuronal population. Enlarged image of stained ACC slice. CaMKII, PV and SOM do not co-localize. (PNG 2291 kb) [file 13041_2015_170_MOESM1_ESM.png]

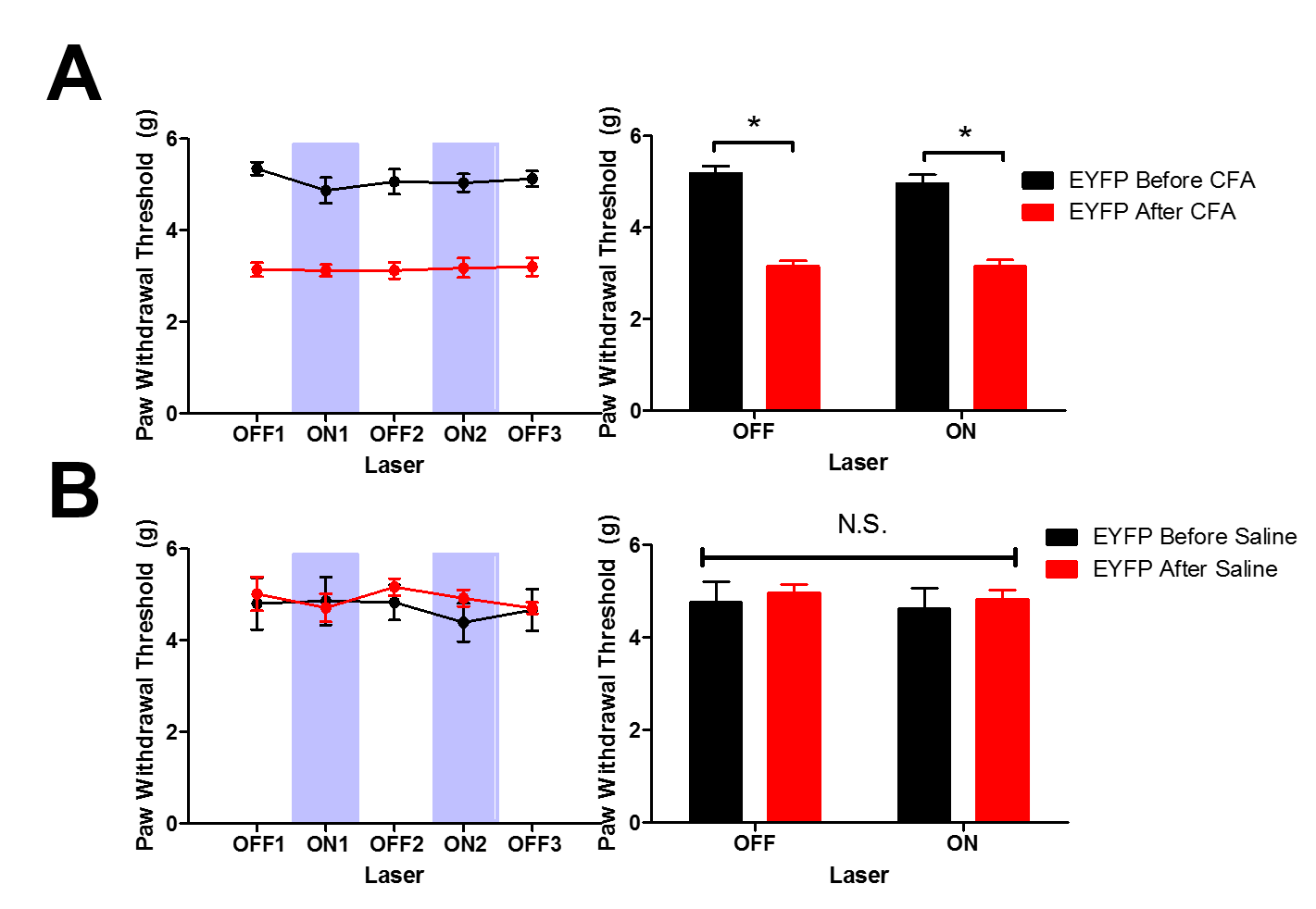

Supplement: Additional file 2: Figure S2. — Control data of optogenetic activation on ACC CaMKII-positive neurons. A, Result of the CaMKII-EYFP-CFA group. There was no light effect during either the pre-test (off: 5.18 ± 0.17 g, on: 4.96 ± 0.19 g; n = 7) or the post-test (off: 3.15 ± 0.12 g, on: 3.15 ± 0.14 g; n = 7). There was only statistically significant effect of CFA treatment per se (p < 0.001, two-way repeated measures ANOVA). B, Result of CaMKII-EYFP-Saline group. There was no light effect either before (off: 4.77 ± 0.44 g, on: 4.63 ± 0.44 g; n = 6) or after saline injection (off: 4.96 ± 0.17 g, on: 4.82 ± 0.22 g; n = 6). (PNG 45 kb) [file 13041_2015_170_MOESM2_ESM.png]

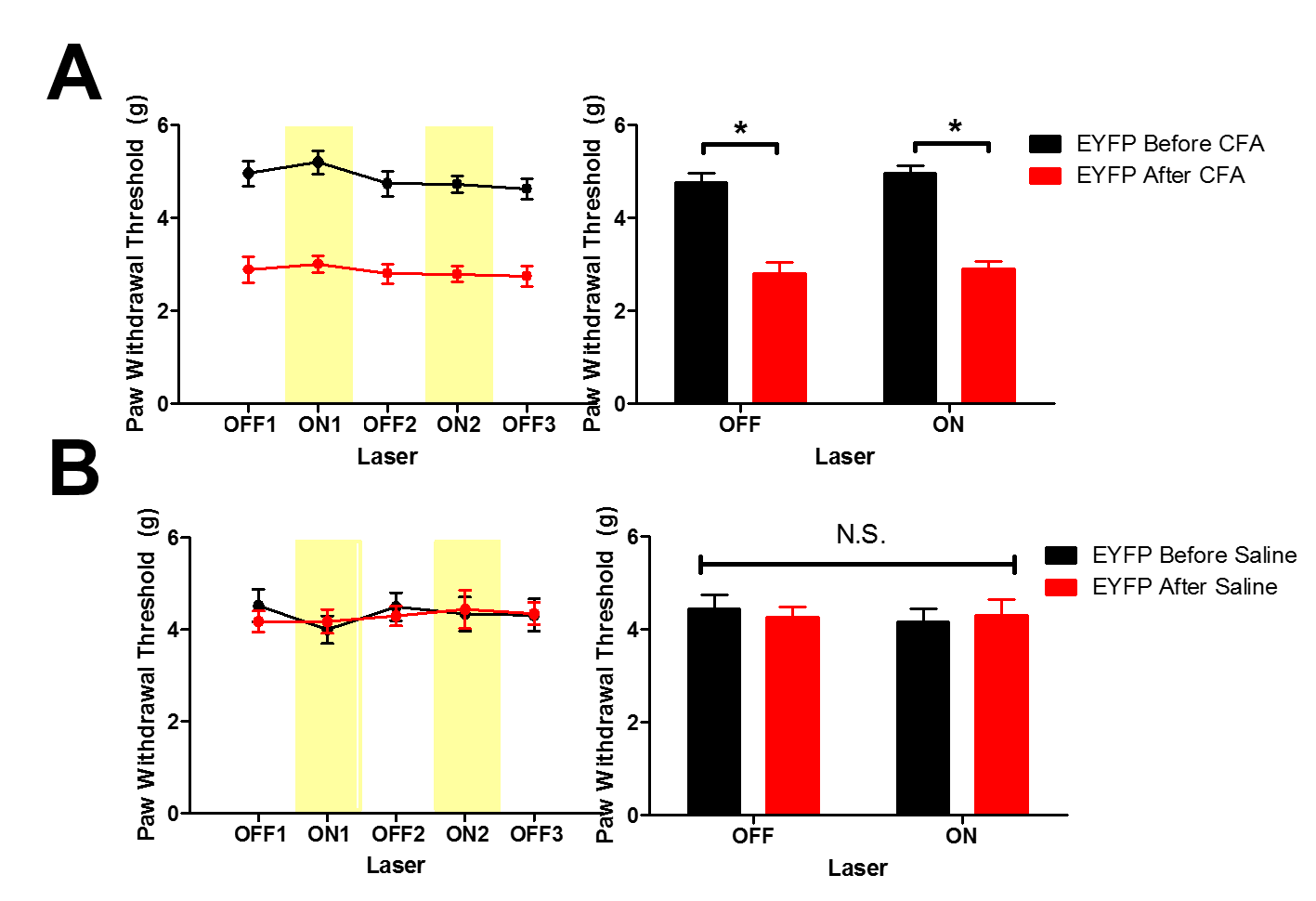

Supplement: Additional file 3: Figure S3. — Control data of optogenetic inhibition on ACC CaMKII-positive neurons. A, Result of the CaMKII-EYFP-CFA group. There was no light effect before (off: 4.77 ± 0.20 g, on: 4.97 ± 0.15 g; n = 6) or after CFA (off: 2.81 ± 0.22 g, on: 2.90 ± 0.16 g; n = 6). There was only a statistically significant effect of CFA treatment per se (p < 0.001, two-way repeated measures ANOVA). B, Result of CaMKII-EYFP-Saline group. There was no light effect either before (off: 4.44 ± 0.30 g, on: 4.17 ± 0.28 g; n = 5) or after CFA (off: 4.27 ± 0.22 g, on: 4.31 ± 0.24 g; n = 5). (PNG 42 kb) [file 13041_2015_170_MOESM3_ESM.png]

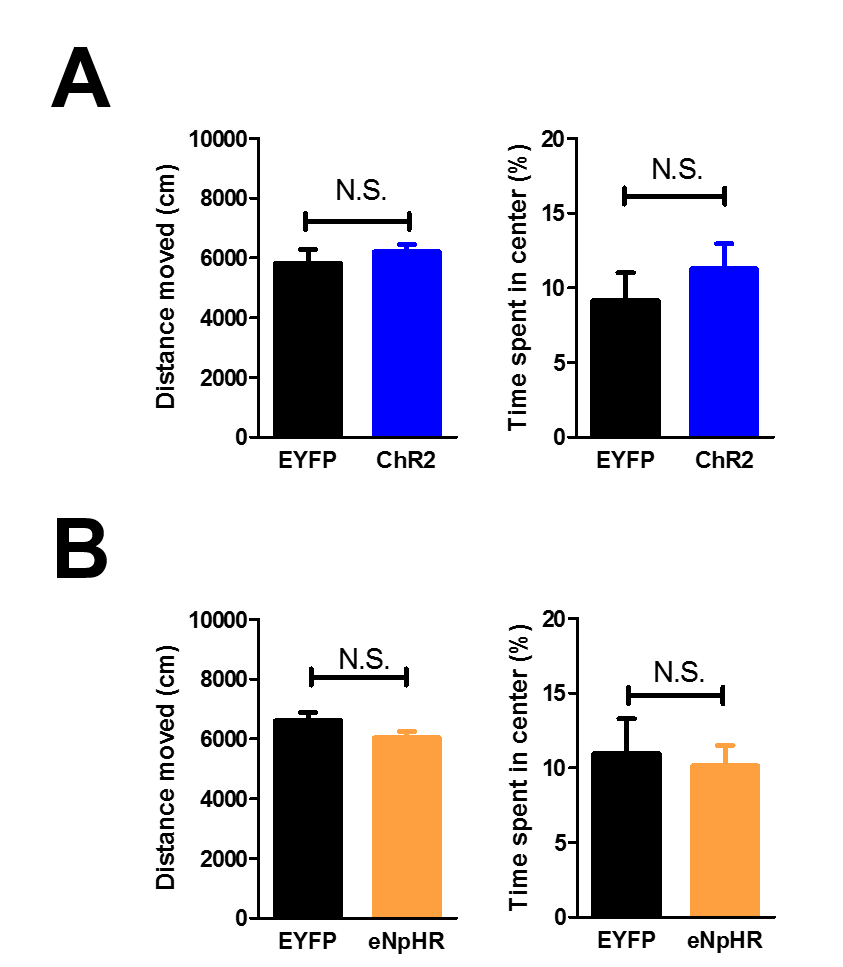

Supplement: Additional file 4: Figure S4. — Control experiments for locomotion and anxiety. A, Results of the CaMKII-EYFP and CaMKII-ChR2 groups in the open field test (OFT). There was no difference between the two groups in either locomotion (EYFP: 5824 ± 457.6 cm, n = 8; ChR2: 6207 ± 244.8 cm, n = 17; p = 0.4273, Unpaired t test) or time spent in the center (EYFP: 9.17 ± 1.85 %, n = 8; ChR2: 11.27 ± 1.68 %, n = 17; p = 0.4559, Unpaired t test). B, Results of the CaMKII-EYFP and CaMKII-eNpHR groups in the OFT. There was no difference between the two groups in either locomotion (EYFP: 6616 ± 278.4 cm, n = 7; eNpHR: 6043 ± 212.9 cm, n = 12; p = 0.1209, Unpaired t test) or time spent in the center (EYFP: 10.96 ± 2.34 %, n = 7; eNpHR: 10.15 ± 1.35 %, n = 12; p = 0.7488, Unpaired t test). (PNG 28 kb) [file 13041_2015_170_MOESM4_ESM.png]

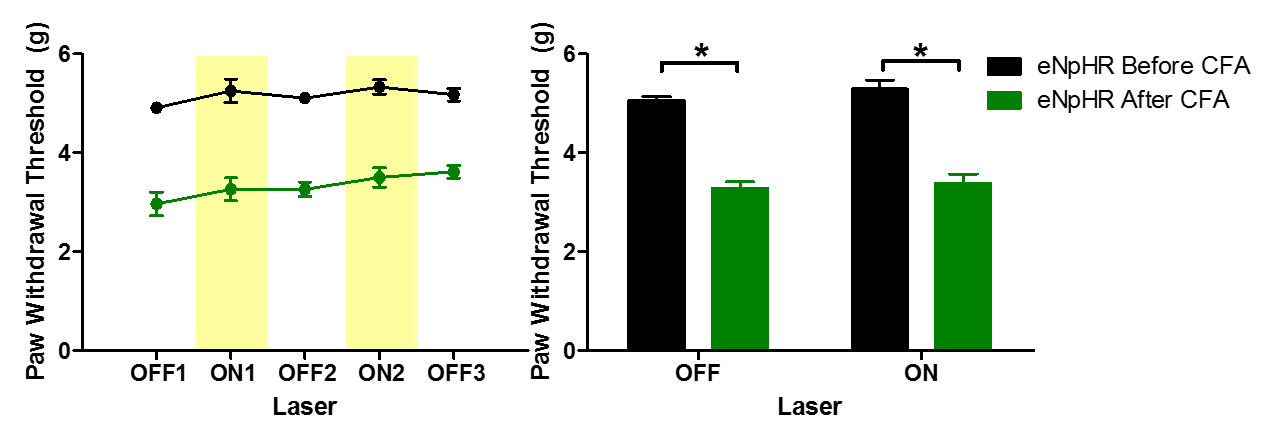

Supplement: Additional file 5: Figure S5. — Optogenetic inhibition of CaMKII-positive neurons in the retrospenial cortex (RSG). There was no light effect either before (off: 5.07 ± 0.07 g, on: 5.29 ± 0.18 g; n = 8) or after CFA injection (off: 3.28 ± 0.13 g, on: 3.38 ± 0.19 g; n = 8). (PNG 21 kb) [file 13041_2015_170_MOESM5_ESM.png]

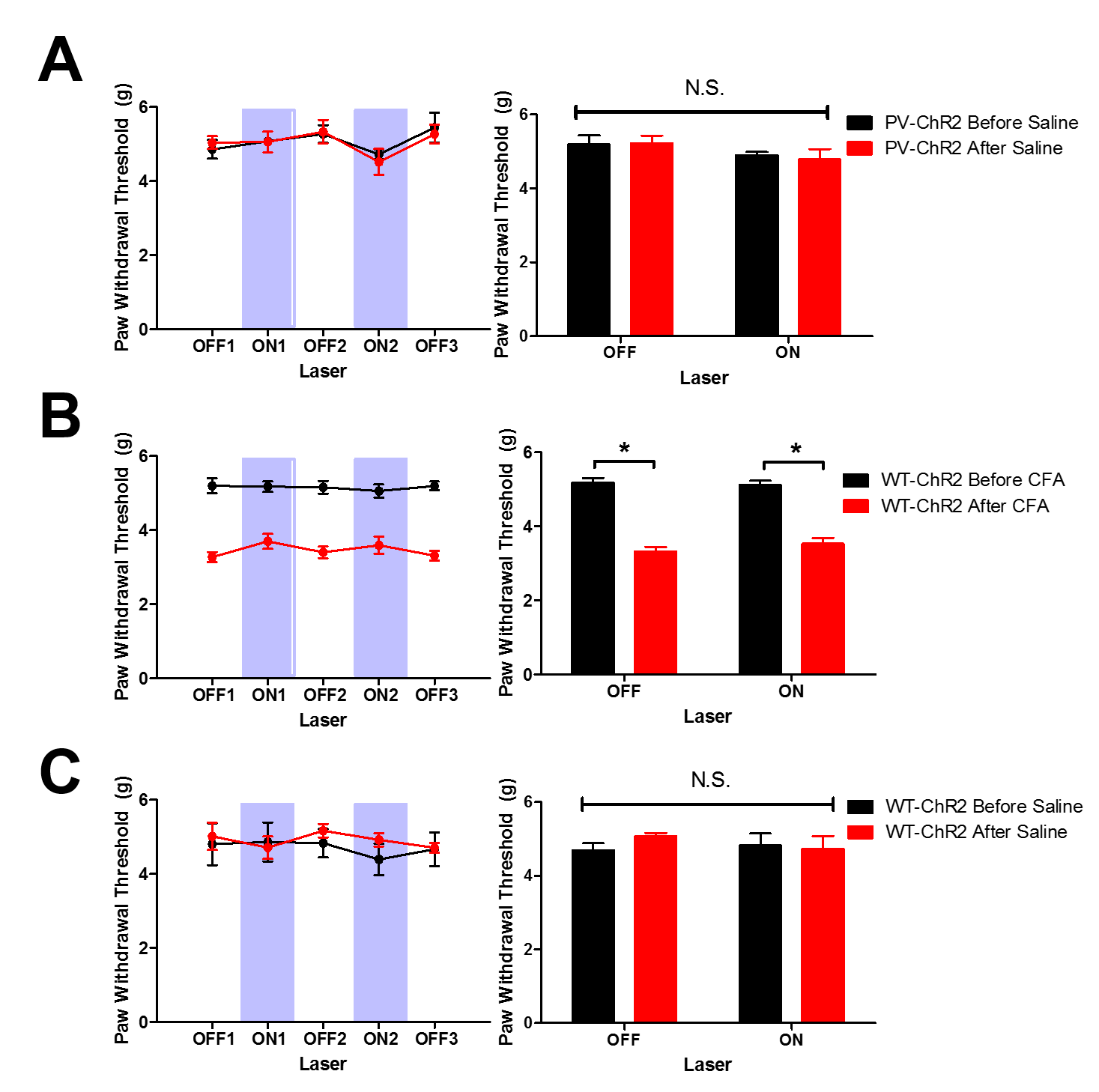

Supplement: Additional file 6: Figure S6. — Control data of optogenetic activation of ACC PV-positive interneurons. A, Result of the PV-ChR2 saline group. There was no light effect either before (off: 5.09 ± 0.22 g, on: 4.84 ± 0.09 g; n = 6) or after saline injection (off: 5.18 ± 0.18 g, on: 4.79 ± 0.23 g; n = 6). B, Result of the WT-ChR2 CFA group. There was no light effect before (off: 5.18 ± 0.13 g, on: 5.12 ± 0.12 g; n = 8) or after CFA (off: 3.33 ± 0.12 g, on: 3.58 ± 0.19 g; n = 8). There was only statistically significant effect of CFA treatment per se (p < 0.001, two-way repeated measures ANOVA). C, Result of the WT-ChR2 saline group. There was no light effect either before (off: 4.7 ± 0.15 g, on: 4.93 ± 0.28 g; n = 5) or after saline (off: 4.94 ± 0.15 g, on: 4.67 ± 0.27 g; n = 5). (PNG 70 kb) [file 13041_2015_170_MOESM6_ESM.png]

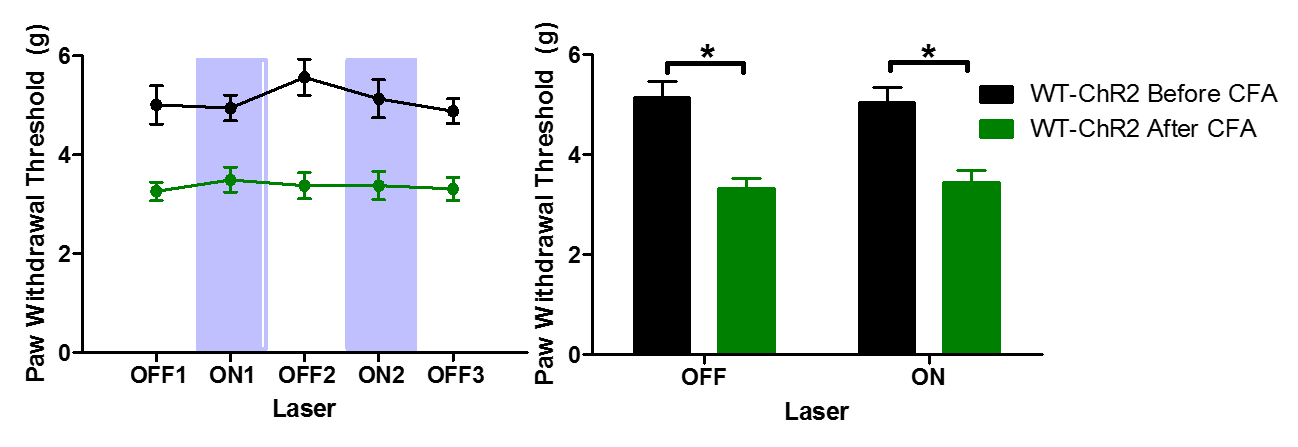

Supplement: Additional file 7: Figure S7. — Control data of optogenetic activation of ACC SOM-positive interneurons. Result of WT-ChR2 CFA group. There was no light effect either before (off: 5.15 ± 0.20 g, on: 5.32 ± 0.20 g; n = 6) or after CFA injection (off: 3.10 ± 0.09 g, on: 3.24 ± 0.07 g; n = 6). (PNG 23 kb) [file 13041_2015_170_MOESM7_ESM.png]
